# Transient contribution of left posterior parietal cortex to cognitive restructuring

**DOI:** 10.1038/srep09199

**Published:** 2015-03-17

**Authors:** Chihiro Sutoh, Daisuke Matsuzawa, Yoshiyuki Hirano, Makiko Yamada, Sawako Nagaoka, Sudesna Chakraborty, Daisuke Ishii, Shingo Matsuda, Haruna Tomizawa, Hiroshi Ito, Hiroshi Tsuji, Takayuki Obata, Eiji Shimizu

**Affiliations:** 1 Department of Cognitive Behavioral Physiology, Graduate School of Medicine, Chiba University, 1-8-1 Inohana, Chuo, Chiba 260-8670, Japan; 2 Research Center for Child Mental Development, Graduate School of Medicine, Chiba University, 1-8-1 Inohana, Chuo, Chiba 260-8670, Japan; 3 Research Center for Charged Particle Therapy, National Institute of Radiological Sciences, 4-9-1 Anagawa, Inage, Chiba 263-8555, Japan; 4 Molecular Imaging Center, National Institute of Radiological Sciences, 4-9-1 Anagawa, Inage, Chiba 263-8555, Japan; 5 National Center for Neurology and Psychiatry, 4-1-1 Ogawa Higashi, Kodaira 187-8551, Japan

## Abstract

Cognitive restructuring is a fundamental method within cognitive behavioural therapy of changing dysfunctional beliefs into flexible beliefs and learning to react appropriately to the reality of an anxiety-causing situation. To clarify the neural mechanisms of cognitive restructuring, we designed a unique task that replicated psychotherapy during a brain scan. The brain activities of healthy male participants were analysed using functional magnetic resonance imaging. During the brain scan, participants underwent Socratic questioning aimed at cognitive restructuring regarding the necessity of handwashing after using the restroom. The behavioural result indicated that the Socratic questioning effectively decreased the participants' degree of belief (DOB) that they must wash their hands. Alterations in the DOB showed a positive correlation with activity in the left posterior parietal cortex (PPC) while the subject thought about and rated own belief. The involvement of the left PPC not only in planning and decision-making but also in conceptualization may play a pivotal role in cognitive restructuring.

When you wash your hands using water, you perceive the water through your senses, and your brain recognises the water for what it is. The human brain tries to understand our outer environment through multisensory routes, namely seeing, hearing, smelling, touching, and tasting. Multisensory integration from early sensory areas to higher-order multisensory regions leads to concept formation as a mental representation in the brain^1^,^2^,^3^. When you see, hear, touch, smell, or taste water, the perceptual experiences stimulate a concept of water that you possess in your brain. Conceptualization enables higher-level conceptual thinking including monitoring, imagining, predicting, hypothesizing, reasoning, planning, evaluating, and decision-making^4^,^5^. After you perceive water and conceptualise what it is in your daily life, you can decide whether you will wash your hands with water or not.

Some people feel distressed when they wash their hands with water due to cognitive dysfunction. For example, obsessive compulsive disorder (OCD) is a psychiatric disorder in which the patient is unable to stop repetitive behaviour such as washing his or her hands in the presence of intrusive thoughts or unpleasant ideas^6^. Many patients with mysophobia (and also some healthy people) have a dysfunctional belief that one will get sick if one does not wash one's hands after using the restroom. When the perception of the threat of illness provoked by an overestimation of the concept of contamination is seen to be greater than the objective degree of the threat, a dysfunctional belief may develop in conjunction with extreme fear and excessive anxiety^7^.

Such dysfunctional beliefs can be changed with psychotherapy. The most effective treatment for OCD is cognitive behavioural therapy, a form of psychotherapy that emphasises the important role of thinking about how we perceive the world and conceptualise it^8^,^9^. The focus of cognitive therapy is changing dysfunctional, extreme and rigid beliefs^10^. Therapists guide patients to develop functional, flexible, self-enhancing beliefs and to react appropriately to the reality of the anxiety-causing situation through a step-by-step process. Cognitive restructuring is a psychotherapeutic process of learning to identify dysfunctional thoughts and develop functional thoughts^11^,^12^; cognitive therapists often use Socratic questioning to facilitate cognitive restructuring. Socratic questioning is a philosophical guiding technique in which Socrates asked his pupils a series of questions in order to challenge the conventional wisdom of his time and to seek truth through dialogue. Examples of Socratic questioning for patients with OCD who cannot stop handwashing include the following: “Can you think of reasons why you should wash your hands?;” “Should you really wash your hands then?;” and “Can you think of reasons why you do not have to wash your hands?” In addition, cognitive therapists often ask their patients to examine their degree of belief (DOB) by asking “How much do you believe that now? Please rate the strength of your belief on a scale of 0–100.”

The learning processes that patients undergo during cognitive restructuring using Socratic questioning may engage neural plasticity to allow the patient to develop flexibility in conceptual thinking. Neuroimaging studies have revealed associations between psychotherapy and changes in the activities of several brain areas including the lateral prefrontal cortex, orbitofrontal cortex, anterior cingulate cortex, striatum, and amygdala^7^. Rather than comparing data acquired before and after psychotherapy, assessing the short-term change in neural activity during psychotherapy could more directly reveal the mechanism of the therapeutic effect. Using functional magnetic resonance imaging (fMRI), we investigated brain activity while healthy individuals performed a special cognitive restructuring task consisting of a series of Socratic questions. The task consisted of three sessions about a common theme, handwashing hygiene (Fig. 1). Two simple picture stimulation sessions that tested dirtiness cognition, namely Sessions I and III, were arranged before and after one cognitive restructuring session based on Socratic questioning (Session II). The questioning session was designed to ask the participants to think about and rate their own beliefs concerning handwashing using four types of questions (Q1, Q2, Q3, and the DOB question).

## Results

### Behavioural results of the cognitive restructuring task

Behavioural results regarding DOB fluctuations showed that the Socratic questioning in the present study effectively induced a trend of attenuation in DOB as Session II proceeded (Fig. 2). DOB scores across all participants showed a significant negative correlation with the number of DOB trials (Spearman's ρ = −0.128; *p* = 0.003). It was revealed that, of the three cognitive restructuring questions (Q1–Q3), Q1 in particular significantly attenuated belief (Supplementary Fig. S1). As an individual index of the wavering in belief, we calculated ΔDOB as the difference between the peak and bottom DOB values (median = 28.0; mean = 31.5; standard deviation = 23.6). For example, one participant rated his DOB as shown by the line plot in Fig. 2, so the ΔDOB between his peak (DOB = 88) and bottom (DOB = 61) values was 27. ΔDOB was then entered into the group analysis of fMRI as a covariate of interest and into the correlation analyses.

### Brain activities during the cognitive restructuring task

We investigated the brain activity accompanying the Socratic questioning during Session II. An analysis using individual ΔDOBs as covariates of interest revealed that changes in DOB showed a positive correlation with activity in the left posterior parietal cortex (PPC) during the three cognitive restructuring questions (Q1–Q3) (brain coordinates: *x*, *y*, *z* = −32, −70, 46; *Z*-score = 4.04; *p* < 0.001; 416 mm^3^; Fig. 3). Due to this result a region of interest (ROI) analysis centred on this peak coordinate was defined and was found to show activity positively correlated with ΔDOB (radius 10 mm; Spearman's ρ = 0.545; *p* = 0.009). On the other hand, no region showed a negative correlation under the same conditions.

In addition, the brain activity observed while the participants rated their DOBs by operating the cursor bar revealed positive correlations between ΔDOB and activity in the left PPC, bilateral supplementary motor cortex, bilateral medial prefrontal cortex, right dorsolateral prefrontal cortex, right middle temporal cortex, and left amygdala, while a negative correlation was found in the left anterior insula (Fig. 4 and Supplementary Table S1). Taken together, these results indicate that the left PPC showed matched brain activity related to belief change while the participants were both considering the Socratic questions and rating DOB.

### Brain activities for picture stimuli

After the cognitive restructuring results from Session II were obtained, we evaluated brain activation contrasts for dirty minus clean toilet pictures. This represented the activation arising from dirtiness perception. This comparison was performed using the data obtained during Sessions I and III. First, the activation in the left posterior parietal ROI defined above was analysed. Individual ΔDOBs were found to be significantly correlated with the difference in regional activities in response to the dirty vs. clean stimuli during Session I (Spearman's ρ = 0.524; *p* = 0.012) and Session III (Spearman's ρ = 0.634; *p* = 0.002) (Supplementary Fig. S2). We then compared brain activations for dirty minus clean toilet pictures during Sessions I and III. Significant differences between the brain activations for these two visual stimuli during Session I were seen in broad areas including the amygdala, supplementary motor area, orbitofrontal cortex, superior parietal lobules, fusiform gyrus, visual cortex, and cerebellum (Supplementary Fig. S2 and Supplementary Table S2). On the other hand, during Session III, significant differences in the comparison of dirty minus clean pictures were found in relatively limited areas such as the lingual gyrus, fusiform gyrus, visual cortex, and cerebellum.

### Correlations between ΔDOB, neuropsychological assessments and questionnaires

To clarify the relationships between task-related cognitive flexibility and individual cognitive ability, correlation coefficients between ΔDOB and the scores obtained from neuropsychological assessments and questionnaires were calculated (Supplementary Table S3). The participants were assessed for intelligence quotient, reasoning ability, set-shifting, and traits for self-recognition of aptitude. However, the results showed no significant correlations of these scores with ΔDOB.

## Discussion

In summary, changing one's belief about the necessity of handwashing in the behavioural result showed a positive correlation with activity in the left PPC while the participants considered the cognitive restructuring questions. This correlation was reproduced in the results from the ROI analysis for the PPC while the participants considered the same questions and from the whole-brain analysis of activity while the participants rated their own DOBs. These results could provide important information for future treatments of local brain areas such as transcranial magnetic stimulation and transcranial direct current stimulation.

The PPC is divided into the superior parietal lobule (SPL) and the inferior parietal lobule (IPL) by the horizontal portion of the intraparietal sulcus. The brain area indicated in the present results to be associated with cognitive restructuring, Brodmann area 7, lies near the border between the SPL and the IPL. A frequently used brain atlas^13^ indicates the brain coordinates of the area as the left SPL while another atlas^14^ indicates the coordinates as the left IPL.

Our pilot approach to clarify neural activity during psychotherapy suggested that a transient activation in the left PPC is associated with cognitive restructuring induced by Socratic questions. As far as the authors know, this is the first brain imaging study to replicate psychotherapy during the scan rather than compare two brain scans before and after psychotherapy. Beyond our expectations, the results suggested the association of the PPC, which is a well-known contributor to sensorimotor transformations. The PPC receives converging inputs from the visual, somatosensory, auditory, and vestibular systems, which use diverse reference frames to encode sensory information^15^,^16^. The human PPC plays a pivotal role in the sensorimotor integration between sensory input and motor output to provide an internal estimate of the state of both the world and one's own body^17^. Additionally, the PPC is now drawing attention due to its role in higher-level cognitive functions, and investigations suggest that it is involved in cognitive functions including abstract reasoning^18^, decision making^19^,^20^, non-first-choice decisions^21^, and action planning^22^. In results similar to those of the present study, activation in this brain region has shown parametrically dependent activity in goal-processing operations^23^. As Llinás argues, thinking may be the evolutionary internalization of movement^24^. Sensorimotor transformation and such higher-level cognitive functions were suggested as candidates to explain how cognitive restructuring processed belief.

The belief change shown in the present study might also be based on concept formation, to which the PPC contributes, although it might also be partially supported by the aforementioned parietal cognitive functions. It is known that damage to the left parietal lobe can cause ideational apraxia, an acquired disorder of motor planning^25^,^26^,^27^. Patients with ideational apraxia show a loss of sensorimotor memories for habitual actions such as washing hands, but without sensory or motor impairments. Additionally, the PPC is known to play a role in cross-modal conceptualization and abstract re-conceptualization, and is hypothesised to play a role in metaphor and the “mirror neuron system^28^,^29^”. Interestingly, cognitive therapists often use metaphor in cognitive behavioural therapy. Our results were congruent with the insight of previous studies that concept formation has a promotional role in cognitive restructuring. If the task were to restrict the participants' way of thinking more, particular function(s) could be distinguished as more dominant contributors to cognitive restructuring. However, the trade-off for such an experimental modification is that the task becomes less similar to what occurs in clinical situations.

The correlation found between the individual ΔDOB and the response in the PPC during cognitive restructuring might explain individual differences in the reactivity to psychotherapy. Participants with wider ideas or deeper thoughts in the belief might induce more activity in the PPC and, thus, have sufficient flexibility to change their beliefs. Various types of functional thinking associated with the PPC such as self-monitoring, imagining, predicting, hypothesizing, reasoning, planning, evaluating, decision-making, and concept formation, in which patients train in psychotherapy, might contribute to this flexibility. Although the DOB question and the cognitive restructuring questions commonly require metacognitive thinking, the former involves several brain areas and the latter involves only the PPC. This might be because the way of thinking is unrestricted; in other words, the unrestricted multimodal thinking in multiple participants in our task might reduce the statistical weight of areas other than the PPC and reveal it as the responsible area. In fact, previous research which tried to predict the therapeutic effect of cognitive behavioural therapy in patients with OCD suggested that activities in many brain regions including the inferior parietal lobule, anterior temporal pole, amygdala, and dorsolateral prefrontal cortex predict the therapeutic effect^30^. The differences in the brain areas identified between previous research and the present research might be based on differences in the task performed (task analogous to psychotherapy or pure symptom provocation with visual stimuli), the imaging schedule (during or before psychotherapy) or the participants (healthy individuals or patients).

The first limitation of the present study was the exclusion of female participants in order to limit the range of the participants' beliefs and the range of their behaviours in imaginative restrooms during brain scan. This limited participation enabled us to exclude a confounding factor related to a gender difference in handwashing behaviour, in which males wash less frequently than females^31^,^32^,^33^. The results from the healthy individuals requires that we be cautious when extrapolating the present results to the context of the mechanisms of psychotherapy for patients, especially as it has been well documented that patients with psychiatric disorders have neural correlates different from those of healthy individuals (e.g., the fear circuit in OCD^34^).

## Methods

### Participants

In this single group, cross-sectional study, we recruited 28 healthy males with normal or corrected-normal vision from among the undergraduate and graduate students of Chiba University through an advertisement for potential participants. The inclusion criteria were (i) right-hand dominance as assessed by the Edinburgh handedness inventory, (ii) fluency in Japanese as a native language, (iii) MRI compatibility (no embedded metal in body, not claustrophobic, not darkness-phobic), (iv) no current psychiatric diagnosis, (v) intelligence quotient > = 80 as assessed by the Wechsler Adult Intelligence Scale-Revised (WAIS-R), and (vi) no current use of psychoactive medications. Six of the 28 potential participants were screened out; one for inclusion criterion (i), one for (iii), one for (v), one due to problems with the MRI machine, and two for sleeping during an MRI scan. Thus, a total of 22 participants were included in the analyses (mean age = 22.1 years; SD = 2.0 years). A summary of the participants' characteristics including the results of neuropsychological assessments and questionnaires is shown in Supplementary Table S3. All participants gave informed consent in accordance with a protocol approved by the Institutional Review Boards of both the Chiba University Graduate School of Medicine and the National Institute of Radiological Sciences.

### Experimental Design

**Figure 1:**
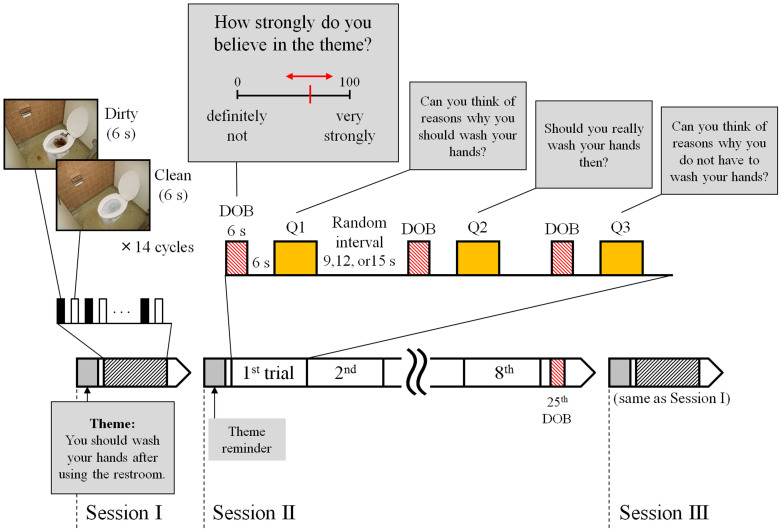
Task designed for functional magnetic resonance imaging (fMRI) to induce cognitive restructuring using Socratic questioning. We designed a task related to a consistent theme (a belief in handwashing) and consisting of three sessions to be performed under fMRI. Using blood oxygen level-dependent (BOLD) responses to the text questions (Q1-Q3) and the degree of belief (DOB) question during Session II, we aimed to identify brain activity correlating with wavering belief. The DOB questions required participants to express their DOB at the time of questioning by pressing a button with the right thumb. Brain activity related to dirtiness cognition was tested during both Sessions I and III by comparing the BOLD responses to the dirty vs. clean stimuli. A theme reminder was presented prior to each session. Photographs were taken by C. S.

**Figure 2:**
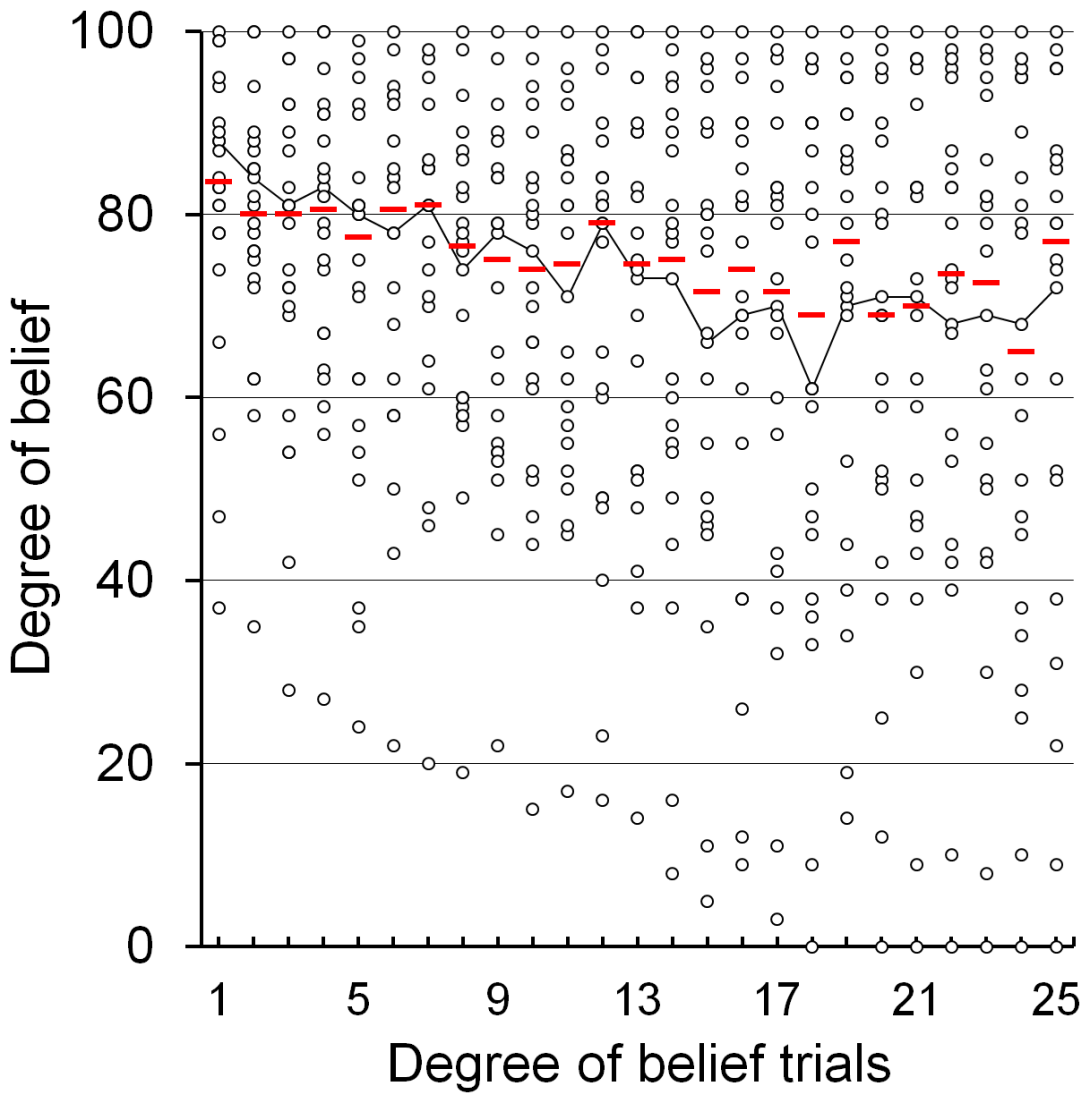
Changes in degree of belief (DOB) in participants during cognitive restructuring session of fMRI task. DOB across all participants showed a trend of attenuation. The DOB scores acquired via the 25 DOB questions for each participant (circles) and the median DOB score of each trial (red bars) are shown here. The line plot is an example of the DOB fluctuation of one participant who rated his ΔDOB as 27, defined as his peak (DOB = 88) minus bottom (DOB = 61) values. A significant negative correlation was found between the number of trials and DOB scores (Spearman's ρ = −0.128; *p* = 0.003).

During the fMRI scanning, we showed all participants a series of visual stimuli projected on a screen in the form of three presentation sessions including pictures and text messages, and asked them to press buttons as part of a task sequence (Fig. 1). In Session I, washing-relevant cue pictures (dirty and clean lavatory bowls) were presented. In Session II, the participants performed a cognitive restructuring task regarding the necessity of handwashing after using the restroom. In Session III, the same washing-relevant cue pictures as those in Session I were presented once again. The sentence “Theme: You should wash your hands after using the restroom” was presented for 21 s prior to each session as the theme reminder. Sessions I, II and III lasted about 5, 15, and 5 minutes, respectively, and the intersession intervals each lasted about 1 minute.

Sessions I and III, which had the same block design, each consisted of an alternating presentation of pictures including dirty and clean toilet bowls in the restroom (clean1 and dirty1 for Session I; clean2 and dirty2 for Session III) and a fixation cross as a baseline. The dirty toilet picture was prepared by contaminating the clean toilet picture with computer-generated excrement. The participants were instructed before the scan to think about what they would think or feel if they had to use the restroom, and how they might use or touch it, while looking at the pictures. A picture presentation sequence consisting of a clean toilet, the fixation, a dirty toilet, and the fixation for 6 s each was repeated 14 times for a total of 336 s.

Session II was designed as a cognitive restructuring task in which participants were visually presented one question in which they were asked to rate their DOB and three cognitive restructuring questions (Q1-Q3) as text messages in a block design. During the DOB question, the text message “How strongly do you believe in the theme?” and a DOB rating scale from 0 (no belief at all; left) to 100 (strongly confident; right) were presented for 6 s. A cursor bar was also shown on the scale and could be moved to the left or right by pressing the corresponding buttons on a keypad. Each participant was repeatedly requested to rate his own DOB by operating the cursor bar. The cursor bar was initially located at a random point on the scale to eliminate bias and to randomise the contributions of motor function to brain activity. Participants controlled the cursor with their right thumb; removing the thumb from the buttons while waiting for the time to expire allowed the question to end. The value to which the cursor pointed at the end of the question time was considered to be the participant's DOB in the theme at that moment. As an index of individual wavering belief, ΔDOB was defined as the difference between the peak and bottom values acquired from the DOB question trials.

Session II also included the following three cognitive restructuring questions (Q1–Q3) presented for 12 s each to induce the participants to simply think about their beliefs regarding handwashing: (Q1) “Can you think of reasons why you should wash your hands?,” (Q2) “Should you really wash your hands then?,” and (Q3) “Can you think of reasons why you do not have to wash your hands?” Sets including one DOB question, a 6-s fixation baseline stimulus, and one of the three cognitive restructuring questions (Q1, Q2, or Q3, chosen in turn) were sequentially presented with a fixation baseline interval for a random duration (9, 12, or 15 s). Three sets constituted one trial. Eight trials and one subsequent DOB question were presented to give a total of 25 DOB questions and 24 nonresponse questions.

### Neuropsychological assessments and questionnaires

To clarify the relationship between task-related cognitive flexibility and individual cognitive ability, all participants underwent the following three neuropsychological assessments and completed two self-reported questionnaires. Intelligence quotient (IQ) was estimated using a three-subtest short form of the WAIS-R made up of the information, letter-number sequencing, and picture completion subtests^35^. As a quantitative assessment of reasoning ability, we assessed the scores achieved in 40 minutes on Raven's advanced progressive matrices, which is a visuospatial non-verbal test independent of cultural background^36^. With the Wisconsin Card Sorting Test (WCST, Keio F-S version^37^), which is a Japanese version of a computer-based test for set-shifting, categories achieved (CA), total error (TE), and perseverative errors of Milner and Nelson (PEM and PEN) were sampled. The self-reported questionnaires, namely the Reflection-Impulsivity Scale^38^ and the Need for Cognition Scale^39^,^40^, were administered to assess the participants' self-recognition about their own aptitude.

### Imaging and statistical analyses

Imaging data were acquired using a 3.0T scanner (Signa; GE Healthcare, Fairfield, CT, USA) with an 8-channel standard head coil. Functional images were obtained by a T2*-weighted echo-planar imaging sequence (repetition time 3000 ms; echo time 30 ms; flip angle 90 degrees; field of view 256 × 256 mm^2^; matrix 64 × 64; interleaved scanning order, 34 slices of 3.8-mm thickness; 0.2-mm gap). A structural scan was then acquired for anatomical referencing using a high-resolution T1-weighted sequence (repetition time 6.776 ms; echo time 1.92 ms; flip angle 12 degrees; field of view 256 × 256 mm^2^; matrix 256 × 256; 160 slices of 1 mm thickness; no gap; inversion time 450 ms). Foam padding was used to minimise the motion of the participant's head during imaging. The task presentation and response recording were computed using Presentation (Neurobehavioral Systems, Inc., Albany, CA, USA). Visual stimuli were presented via a back-projected screen that could be seen via a mirror placed in front of the participant's eyes.

A statistical parametric mapping (SPM) program (SPM8; Wellcome Trust Centre for Neuroimaging, London, UK) was used for image preprocessing and statistical analyses. Each of the participants' sequential functional volumes was corrected for slice timing and then realigned to the first scan for motion correction. Subsequently, these functional images were spatially normalised with the Montreal Neurological Institute template echo planner image. Normalised images were resampled into 2 × 2 × 2 mm^3^ voxels. Finally, the images were smoothed with a 6-mm full-width half-maximum Gaussian kernel.

Preprocessed images were entered into a general linear model^41^ that modelled the canonical hemodynamic response functions convolved with a boxcar representing the task conditions. The following task conditions were modelled as regressors (explanatory variables) in the design matrix: the DOB question, Q1, Q2, Q3, clean1, dirty1, clean2, and dirty2. Six movement parameters (translation and rotation in x, y, and z) resulting from the realignment process were also included as potential covariates of no interest. We constructed whole-brain statistical parametric maps representing the association between the observed BOLD signal and regressors for each participant.

A whole-brain, voxel-wise random effect analysis was used to test the effects of such conditions on BOLD signals. For the first-level individual analysis, the effect of each experimental condition was convolved. Contrasts of dirty minus clean conditions within Sessions I and III, a contrast of the global effects of the three nonresponse questions (Q1, Q2, and Q3), and a contrast of the DOB question were also calculated at this level. A second-level group analysis was then computed with one sample t-test on each contrast. To explore the brain regions potentially relating to the effect of cognitive restructuring, individual ΔDOBs were included as covariates of interest, particularly in the analysis of contrasts in the global effects of the three nonresponse questions and of the contrast of the DOB question. Clusters surviving a peak-level threshold of uncorrected *p* < 0.001 and a voxel extension threshold of 10 consecutive voxels (80 mm^3^) were reported in the results of the whole-brain analysis^42^. Montreal Neurological Institute coordinates of the clusters in the fMRI results were nonlinearly transformed into the Talairach coordinate space^43^ and then labelled by the Talairach Client^13^.

**Figure 3:**
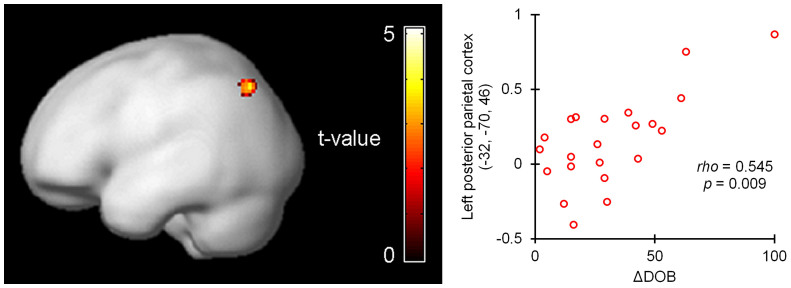
Positive correlation between activity in left posterior parietal cortex during thinking about cognitive restructuring questions and individual index of wavering in belief (ΔDOB). Activity in the left posterior parietal cortex during the three cognitive restructuring questions showed a positive correlation with individual ΔDOB. fMRI demonstrated a statistical parametric t map of the acquired cluster of activity rendered onto a smoothed brain template (k = 52; *p* < 0.001). The plot represents the relationship between activity in the spherical region of interest including the left posterior parietal cortex and ΔDOB (% signal change; 10-mm radius centred on x, y, z = −32, −70, 46 of the Montreal Neurological Institute brain coordinates; Spearman's ρ = 0.545, *p* = 0.009).

**Figure 4:**
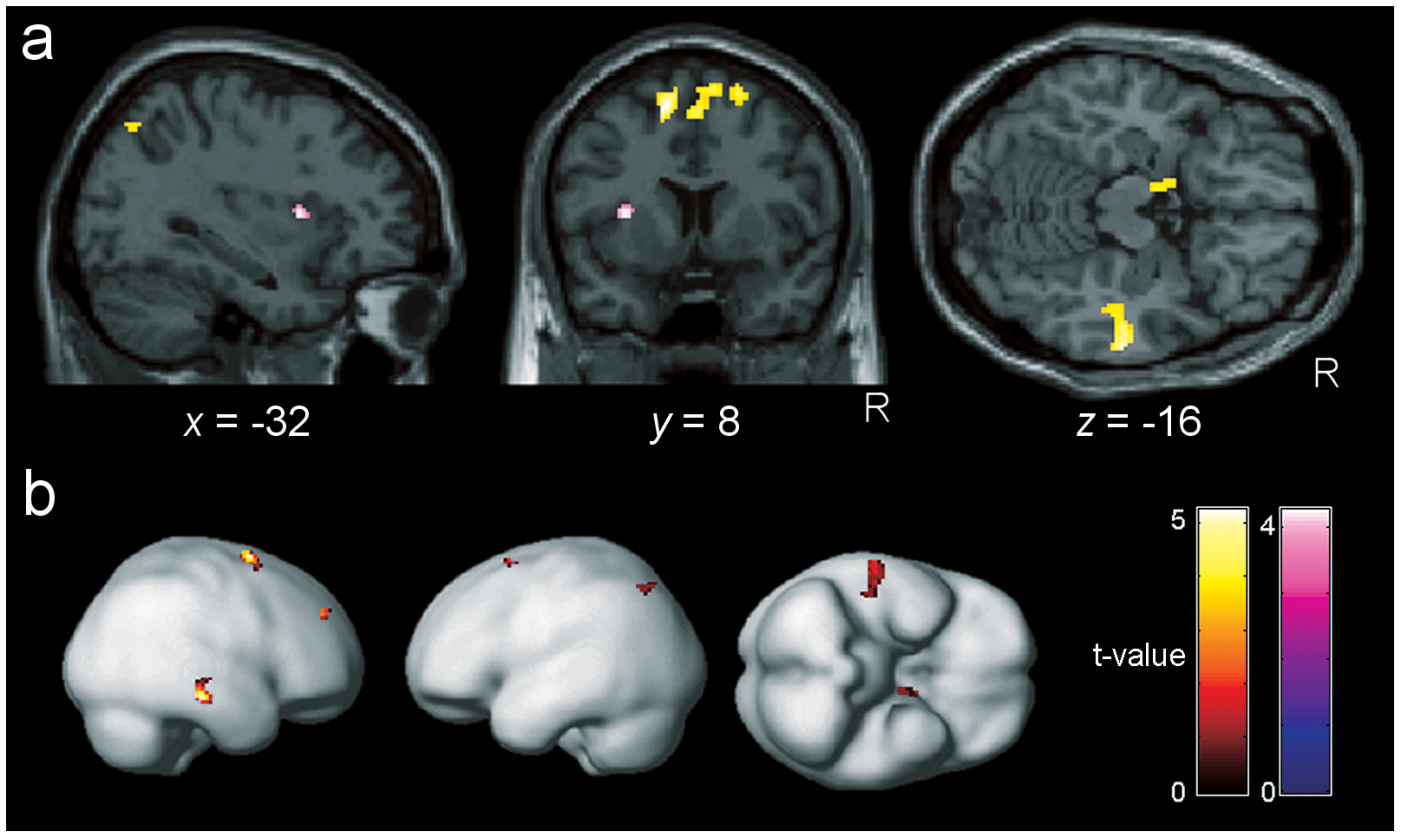
Brain areas in which activity while rating one's own degree of belief correlated with the individual index of wavering in belief (ΔDOB). Brain areas showed activity correlating with individual ΔDOB while the subjects answered the DOB questions. In parallel with its activity during the three cognitive restructuring questions (Fig. 3), the left posterior parietal cortex showed a positive correlation with ΔDOB. Clusters that survived the height threshold of *p* < 0.001, uncorrected, and an extent threshold of 10 voxels are rendered on the sections (a) and on a smoothed brain template (b). Positive correlations with ΔDOB (red to yellow) were found in the bilateral supplementary motor cortex, bilateral medial prefrontal cortex, right dorsolateral prefrontal cortex, right middle temporal cortex, and left amygdala, and a negative correlation (blue to purple) was found in the left anterior insula.

Following this group analysis, an ROI analysis was performed to evaluate the functional relationship between brain activity and wavering belief. Due to the results of the global effects of the three nonresponse questions (Fig. 3), a spherical ROI with a radius of 10 mm centred on the Montreal Neurological Institute (MNI) brain coordinates x, y, z = −32, −70, 46 was defined using the MarsBaR toolbox 0.43 for SPM^44^. Mean percent signal changes in the ROI were calculated for each contrast of the three questions (global) and the DOB question.

Correlation coefficients (Spearman's ρ) were calculated between the DOB scores across all participants and the number of DOB trials, between ΔDOB and BOLD signal changes in the ROI, and between ΔDOB and the scores from neuropsychological assessments. To evaluate the changing effects of the three cognitive restructuring questions on DOB, repeated measured two-way analysis of variance (ANOVA) was performed on the rank-transformed DOB scores^45^,^46^. Two factors were assigned in this two-way ANOVA: before and after each question, and eight trials for each cognitive restructuring question. We used SPSS 12.0 (SPSS, Inc., Chicago, IL, USA) for these correlation analyses and two-way ANOVA, and considered *p* < 0.05 to be statistically significant.

## Author Contributions

C.S., D.M., M.Y., S.N., T.O. and E.S. designed conceptual study design. C.S., D.M., Y.H. and T.O. designed the fMRI task. C.S., D.M., Y.H., S.C. and T.O. obtained MRI data. D.I., S.M. and H.T. obtained clinical data. C.S., D.M. and Y.H. analysed all data. C.S., D.M., Y.H. and T.O. interpreted results. C.S. and E.S. drafted the manuscript. All authors were involved in the revisions. H.I., H.T. and O.T. coordinated the study and secured the funding.

## Supplementary Material

Supplementary Information Supplementary Information
