# Supplementary material for: Transient contribution of left posterior parietal cortex to cognitive restructuring
Source: Sci Rep. 2015 Mar 17;5:9199. doi: 10.1038/srep09199 (PMC4361861; doi:10.1038/srep09199)
Supplement: Supplementary Information [file srep09199-s1.doc]

Supplementary Information for

Transient contribution of left posterior parietal cortex to cognitive restructuring

Chihiro Sutoh, Daisuke Matsuzawa, Yoshiyuki Hirano, Makiko Yamada, Sawako Nagaoka, Sudesna Chakraborty, Daisuke Ishii, Shingo Matsuda, Haruna Tomizawa, Hiroshi Ito, Hiroshi Tsuji, Takayuki Obata, Eiji Shimizu

**Supplementary Figure S1. Effect of three cognitive restructuring questions on degree of belief (DOB)**

Change in DOB was defined as the DOB score after a question minus the DOB score before the same question, and then change in DOB was averaged for each individual. A significant attenuating effect was found for Q1 (*F*(1, 21) = 12.251; *p* = 0.002; partial *η*^2^ = 0.368) as the main effect by rank-transformed two-way analysis of variance and Bonferroni’s multiple comparisons†, but not for Q2 (*F*(1, 21) = 0.709; *p* = 0.409; partial *η*^2^ = 0.033) or Q3 (*F*(1, 21) = 5.529; *p* = 0.029; partial *η*^2^ = 0.208). Circles represent outliers.


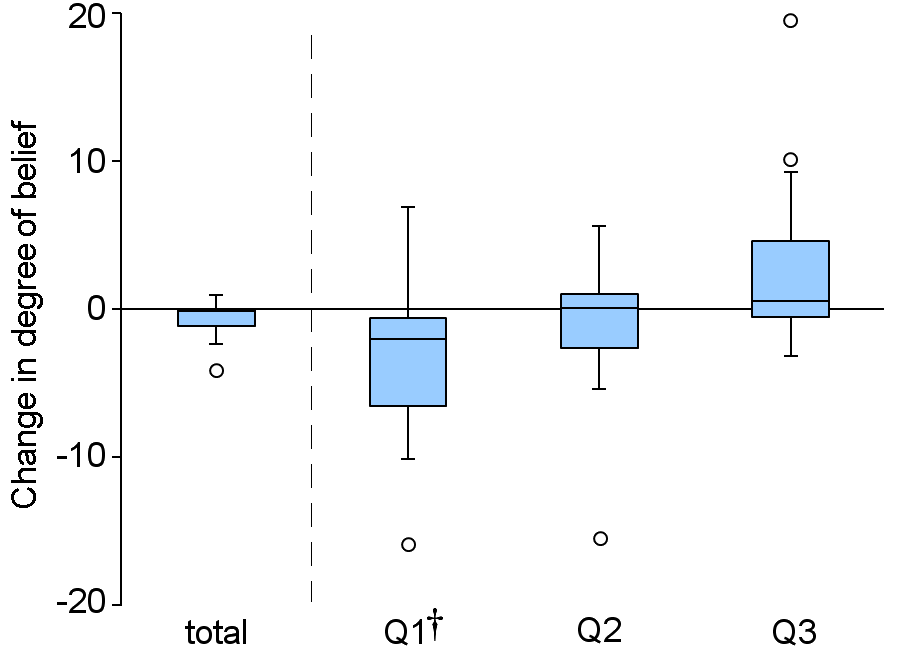


**Supplementary Figure S2.** **Brain activation associated with dirtiness cognition shown by comparison between activities in response to dirty minus clean stimuli, during Sessions I and III**

Upper and lower are the fMRI results during Sessions I and III respectively. (**a**) Relationships between differential activity in response to the dirty minus clean stimuli in the left posterior parietal cortex and ΔDOB are plotted. Individual ΔDOBs were significantly correlated with regional activity in response to the dirty vs. clean stimuli during Sessions I (Spearman’s rho = 0.524; *p* = 0.012) and III (Spearman’s rho = 0.634; *p* = 0.002). (**b**) Amygdala responses associated with dirtiness cognition are shown. During Session I, a comparison of blood oxygen level-dependent (BOLD) responses to the dirty pictures minus those to the clean pictures revealed significant differential activity in the bilateral amygdala (x, y, z = -24, -2, -26; Z-score = 4.26; *p* < 0.001; 2592 mm^3^; x, y, z = 34, -4, -28; Z-score = 3.50; *p* < 0.001; 1072 mm^3^). For the same comparison during Session III, BOLD in the bilateral amygdala showed no differential activity exceeding the threshold.

**Supplementary Table S1. Brain areas that showed correlated activity while answering the DOB questions with individual ΔDOB (graphically illustrated in Fig. 4)**

|  | ***H*** | ***Label*** | ***Voxels*** | ***Volume*** | ***BA*** | ***Coordinates*** | | | ***Z*** |
| --- | --- | --- | --- | --- | --- | --- | --- | --- | --- |
|  |  |  |  | ***(mm^3^)*** |  | ***x*** | ***y*** | ***z*** |  |
| Positive correlation | | |  |  |  |  |  |  |  |
|  | L | Medial Frontal Gyrus | 86 | 688 | 6 | -14 | 8 | 56 | 4.10 |
|  | L | Medial Frontal Gyrus | 20 | 160 | 6 | -8 | 28 | 40 | 3.42 |
|  | R | Superior Frontal Gyrus | 147 | 1176 | 6 | 20 | 2 | 68 | 3.83 |
|  | R | Superior Frontal Gyrus | 24 | 192 | 9 | 28 | 50 | 34 | 3.56 |
|  | R | Middle Temporal Gyrus | 148 | 1184 | 21 | 64 | -22 | -16 | 3.95 |
|  | L | Superior Parietal Lobule | 19 | 152 | 7 | -30 | -72 | 50 | 3.33 |
|  | L | Amygdala - Hypothalamus | 19 | 152 |  | -8 | -6 | -16 | 3.39 |
| Negative correlation | | |  |  |  |  |  |  |  |
|  | L | Insula | 20 | 160 |  | -32 | 8 | 10 | 3.55 |

Abbreviations: H, hemisphere; BA, Brodmann area. Number of activated voxels that survived the height threshold of *p* < 0.001, uncorrected, and an extent threshold of 10 voxels.

**Supplementary Table S2. Brain areas that showed significant differences between activities in response to dirty and clean visual stimuli during Sessions I and III**

|  |  | ***Voxels*** | ***Volume*** | ***b*** | ***Label*** | ***BA*** | ***Coordinates*** | | | ***Z*** |
| --- | --- | --- | --- | --- | --- | --- | --- | --- | --- | --- |
|  |  |  | ***(mm^3^)*** |  |  |  | ***x*** | ***y*** | ***z*** |  |
| Dirty - Clean in Session I | | |  |  |  |  |  |  |  |  |
|  | Limbic Lobe | 324 | 2592 | L | Uncus | 28 | -30 | 4 | -24 | 4.28 |
|  |  |  |  | L | Amygdala |  | -24 | -2 | -26 | 4.26 |
|  |  | 134 | 1072 | R | Uncus | 28 | 28 | 4 | -26 | 4.22 |
|  |  |  |  | R | Amygdala |  | 34 | -4 | -28 | 3.50 |
|  |  | 119 | 952 | L | Parahippocampal Gyrus | 35 | -24 | -26 | -16 | 4.15 |
|  |  | 24 | 192 | L | Parahippocampal Gyrus | 28 | -22 | -14 | -14 | 3.64 |
|  | Frontal Lobe | 10 | 80 | L | Superior Frontal Gyrus | 6 | -6 | 18 | 70 | 3.33 |
|  |  | 306 | 2448 | R | Superior Frontal Gyrus | 6 | 4 | 16 | 68 | 3.68 |
|  |  |  |  | R | Medial Frontal Gyrus | 6 | 4 | 2 | 66 | 3.74 |
|  |  | 29 | 232 | R | Medial Frontal Gyrus | 6 | 2 | -18 | 68 | 3.43 |
|  |  | 40 | 320 | L | Medial Frontal Gyrus | 8 | -6 | 50 | 48 | 3.79 |
|  |  | 60 | 480 | L | Middle Frontal Gyrus | 6 | -22 | -10 | 66 | 3.64 |
|  |  | 22 | 176 | R | Inferior Frontal Gyrus | 47 | 30 | 26 | -22 | 3.93 |
|  |  | 28 | 224 | L | Inferior Frontal Gyrus | 47 | -24 | 34 | -8 | 3.70 |
|  |  | 41 | 328 | R | Inferior Frontal Gyrus | 47 | 30 | 32 | -10 | 3.67 |
|  | Temporal Lobe | 36 | 288 | L | Fusiform Gyrus | 37 | -40 | -48 | -20 | 3.40 |
|  | Parietal Lobe | 17 | 136 | L | Superior Parietal Lobule | 7 | -18 | -68 | 66 | 3.60 |
|  |  | 29 | 232 | L | Superior Parietal Lobule | 7 | -28 | -60 | 48 | 3.34 |
|  |  | 219 | 1752 | R | Superior Parietal Lobule | 7 | 26 | -66 | 50 | 3.77 |
|  |  |  |  | R | Precuneus | 7 | 12 | -66 | 52 | 3.79 |
|  |  | 80 | 640 | R | Precuneus | 7 | 20 | -82 | 36 | 3.62 |
|  |  | 19 | 152 | L | Precuneus | 7 | -18 | -78 | 50 | 3.22 |
|  | Occipital Lobe | 1663 | 13304 | R | Inferior Occipital Gyrus | 17 | 20 | -94 | -14 | 5.29 |
|  |  | 1653 | 13224 | L | Lingual Gyrus | 18 | -18 | -84 | -20 | 4.68 |
|  |  | 56 | 448 | L | Middle Occipital Gyrus | 19 | -26 | -84 | 10 | 3.52 |
|  | Cerebellum | 11 | 88 | L | Anterior Lobe |  | -10 | -42 | -36 | 3.41 |
|  |  | 16 | 128 | R | Anterior Lobe |  | 12 | -38 | -36 | 3.59 |
|  |  |  |  |  |  |  |  |  |  |  |
| Dirty - Clean in Session III | | |  |  |  |  |  |  |  |  |
|  |  | 833 | 6664 | L | Lingual Gyrus | 18 | -16 | -90 | -16 | 4.48 |
|  |  |  |  | L | Fusiform Gyrus | 19 | -22 | -84 | -16 | 4.44 |
|  |  |  |  | L | Posterior Lobe (Cerebellum) |  | -24 | -82 | -26 | 4.10 |
|  |  | 351 | 2808 | R | Lingual Gyrus | 17 | 18 | -96 | -10 | 4.02 |
|  |  |  |  | R | Middle Occipital Gyrus | 18 | 30 | -84 | -4 | 3.69 |
|  |  |  |  | R | Posterior Lobe (Cerebellum) |  | 22 | -84 | -24 | 3.66 |

Abbreviations: H, hemisphere; BA, Brodmann area. Number of activated voxels that survived the height threshold of p < 0.001, uncorrected, and an extent threshold of 10 voxels.

**Supplementary Table S3. Demographics of participants and results of neuropsychological assessments, and their correlation coefficients with index of wavering in belief (ΔDOB)**

|  |  |  |  |  | ***Correlation***  ***with ΔDOB*** | |
| --- | --- | --- | --- | --- | --- | --- |
|  |  | ***Mean*** | ***SD*** |  | ***rho*** | ***p*** |
| Age |  | 22.1 | 2.0 |  | -0.243 | 0.276 |
| Intelligence quotient | | 102.0 | 14.1 |  | 0.000 | 0.989 |
| RAPM |  | 29.9 | 3.6 |  | 0.219 | 0.328 |
| WCST | CA | 5.3 | 0.7 |  | 0.100 | 0.656 |
|  | TE | 11.3 | 2.5 |  | -0.289 | 0.191 |
|  | PEM | 1.0 | 1.6 |  | -0.200 | 0.372 |
|  | PEN | 1.4 | 1.6 |  | 0.030 | 0.894 |
| RIS |  | 28.1 | 4.8 |  | -0.180 | 0.422 |
| NFS |  | 69.5 | 12.1 |  | -0.220 | 0.324 |

Abbreviations: DOB, degree of belief; RAPM, Raven’s advanced progressive matrices; WCST, Wisconsin Card Sorting Test; CA, categories achieved; TE, total error; PEM, perseverative errors of Milner ; PEN, perseverative errors of Nelson; RIS, Reflection-Impulsivity Scale; NFS, Need for Cognition Scale .
